# Prospective Antiretroviral Treatment of Asymptomatic, HIV-1 Infected Controllers

**DOI:** 10.1371/journal.ppat.1003691

**Published:** 2013-10-10

**Authors:** Hiroyu Hatano, Steven A. Yukl, April L. Ferre, Erin H. Graf, Ma Somsouk, Elizabeth Sinclair, Mohamed Abdel-Mohsen, Teri Liegler, Kara Harvill, Rebecca Hoh, Sarah Palmer, Peter Bacchetti, Peter W. Hunt, Jeffrey N. Martin, Joseph M. McCune, Russell P. Tracy, Michael P. Busch, Una O'Doherty, Barbara L. Shacklett, Joseph K. Wong, Steven G. Deeks

**Affiliations:** 1 Department of Medicine, University of California San Francisco, San Francisco, California, United States of America; 2 Department of Medicine, San Francisco Veterans Affairs Medical Center, San Francisco, California, United States of America; 3 Department of Medical Microbiology and Immunology, School of Medicine, University of California, Davis, Davis, California, United States of America; 4 Department of Pathology and Laboratory Medicine, University of Pennsylvania, Philadelphia, Pennsylvania, United States of America; 5 Karolinska Institutet and Swedish Institute for Infectious Disease Control, Solna, Sweden; 6 Centre for Virus Research, Westmead Millennium Institute and University of Sydney, Westmead, Australia; 7 Department of Epidemiology and Biostatistics, University of California San Francisco, San Francisco, California, United States of America; 8 Departments of Pathology and Biochemistry, University of Vermont, Colchester, Vermont, United States of America; 9 Blood Systems Research Institute, San Francisco, California, United States of America; 10 Department of Laboratory Medicine, University of California San Francisco, San Francisco, California, United States of America; Emory University, United States of America

## Abstract

The study of HIV-infected “controllers” who are able to maintain low levels of plasma HIV RNA in the absence of antiretroviral therapy (ART) may provide insights for HIV cure and vaccine strategies. Despite maintaining very low levels of plasma viremia, controllers have elevated immune activation and accelerated atherosclerosis. However, the degree to which low-level replication contributes to these phenomena is not known. Sixteen asymptomatic controllers were prospectively treated with ART for 24 weeks. Controllers had a statistically significant decrease in ultrasensitive plasma and rectal HIV RNA levels with ART. Markers of T cell activation/dysfunction in blood and gut mucosa also decreased substantially with ART. Similar reductions were observed in the subset of “elite” controllers with pre-ART plasma HIV RNA levels below conventional assays (<40 copies/mL). These data confirm that HIV replication persists in controllers and contributes to a chronic inflammatory state. ART should be considered for these individuals (ClinicalTrials.gov NCT01025427).

## Author Summary

HIV-infected “controllers” are rare individuals who are HIV-seropositive but are able to maintain low levels of plasma HIV RNA in the absence of antiretroviral therapy (ART). There has been intense interest in characterizing these unique individuals because they have been considered as a potential model for a “functional cure” of HIV. Previously, our group has shown that controllers have elevated levels of T cell activation and accelerated atherosclerosis, suggesting that very low levels of viral replication may lead to disproportionately high levels of immune activation. However, the degree to which viral replication contributes to these outcomes is not known. We therefore conducted the first, prospective study of ART initiation in a cohort of asymptomatic HIV-infected controllers, in order to determine the virologic and immunologic effects of treating controllers with ART. Controllers had a significant decreases in ultrasensitive plasma HIV RNA, rectal HIV RNA, and markers of T cell activation/dysfunction in blood and gut mucosa with ART. Similar reductions were observed in the subset of “elite” controllers with extremely low pre-ART plasma HIV RNA levels (<40 copies/mL). These data suggest that HIV replication persists in controllers and contributes to a chronic inflammatory state.

## Introduction

HIV-infected “controllers” are individuals who are HIV-seropositive but are able to maintain low levels of plasma HIV RNA in the absence of antiretroviral therapy (ART) [1]. These individuals are rare, comprising less than 1–7% of the HIV-infected population, depending upon the plasma HIV RNA criteria that are used to define the group [2], [3], [4]. Most controllers have evidence of strong host immune responses, which have been widely assumed to be responsible for durable viral control. Because knowledge regarding these protective immune responses might lead to novel interventions aimed at preventing or curing HIV infection, there has been intense interest in further characterizing these unique individuals.

Multiple groups have examined how HIV is controlled by these individuals [5], [6], 7,8,9. More recently, our group has focused on defining the potential clinical consequences of long-term, host-mediated, virologic control. We and others have shown that: (1) the vast majority of controllers have stable low-level viremia [10], [11]; (2) controllers have elevated levels of microbial translocation and T cell activation compared to HIV-negative and ART-suppressed individuals [12], [13]; (3) a minority (7–10%) of controllers with high levels of T cell activation progress immunologically to AIDS despite preservation of virologic control [12]; and (4) controllers have accelerated measures of atherosclerosis compared to HIV-negative individuals, even after adjustment for traditional cardiovascular risk factors [14], [15]. Collectively, these data suggest that very low levels of viral replication may lead to disproportionately high levels of immune activation in HIV-infected controllers, which may lead to an increased risk of AIDS- and non-AIDS defining events. However, the degree to which viral replication contributes to these outcomes is not known. No prospective ART studies have been performed in controllers, because it has generally been assumed that most controllers do not need ART due to their ability to control plasma viremia to very low levels.

We therefore conducted the first, prospective study of antiretroviral therapy in a cohort of asymptomatic HIV-infected controllers, in order to determine the virologic and immunologic effects of treating controllers with ART. We also measured changes in biomarkers of inflammation and coagulation. Multiple biomarkers (e.g., high sensitivity C-reactive protein and D-dimer) remain elevated in both untreated and treated non-controllers [16], and have been shown to be strongly predictive of morbidity and all-cause mortality in ART-treated non-controllers [17], [18], [19]. We therefore examined whether ART initiation led to a reduction in biomarkers of inflammation and coagulation in controllers, in order to assess whether low-level viral replication has any potential immunologic and clinical consequences in these individuals.

## Results

### Study Participants

Sixteen asymptomatic controllers were prospectively treated with open-label raltegravir+tenofovir/emtricitabine for 24 weeks. Controllers were defined by the following inclusion criteria: (1) HIV-seropositive; (2) ART untreated; and (3) plasma HIV RNA <1,000 copies/mL for ≥12 months. Exclusion criteria included: (1) known rheumatologic conditions (e.g., systemic lupus erythematosus), because of the potential for biologic false-positive testing on HIV antibody tests; (2) known kidney disease; (3) known bone disease, including pathologic fractures; (4) chronic hepatitis B infection, because of the potential risk of liver abnormalities after starting and stopping tenofovir/emtricitabine in patients with chronic hepatitis B infection; (5) serious illness requiring hospitalization or parental antibiotics within the preceding 3 months; and (6) pregnant or breastfeeding women.

Subjects were seen every four weeks. Plasma and peripheral blood mononuclear cells (PBMCs) were collected and detailed interviews were conducted at the majority of visits. Thirteen out of 16 subjects consented to undergo 3 serial colorectal biopsies at weeks −2, 6, and 22. Five out of 16 subjects also underwent leukapheresis at weeks −4 and week 21 in order to obtain large PBMC samples for measurement of integrated HIV DNA. Adherence to study drug was measured at every study visit by self-report and pill-count. An independent Data Monitoring Committee comprised of three individuals from the scientific community met at 12, 24, 48, and 60 weeks after the enrollment of the first subject, and at 60 weeks after the enrollment of the last subject.

### Baseline Characteristics (Table 1)

**Table 1:** Baseline characteristics (n = 16).

Baseline Characteristic	Median	IQR
Age (years)	49	(40–56)
Gender	14 male, 2 female	
CD4+ T cell count (cells/mm^3^)	616	(476–801)
CD8+ T cell count (cells/mm^3^)	897	(623–1434)
CD4+:CD8+ T cell count ratio	0.71	(0.51–0.95)
Nadir CD4+ T cell count (cells/mm^3^)	590	(458–746)
Plasma HIV-1 RNA, <40 copy/mL assay (copies/mL)	77	(40–324)
Number of subjects with plasma HIV-1 RNA <40 copies/mL	4/16	
Plasma HIV-1 RNA, <0.3 copy/mL assay (copies/mL)	23	(0.3–175)
Duration of HIV diagnosis (years)	10	(4.5–24)
Number of subjects with chronic hepatitis C virus infection^a^	3	

Data represent medians and interquartile ranges (IQR) unless otherwise noted.

a At baseline, 3 subjects had chronic hepatitis C infection; 2 additional subjects had spontaneously cleared hepatitis C more than 2 years prior to baseline, and 1 additional subject had completed successful treatment with pegylated interferon and ribavirin more than 1 year prior to baseline.

All subjects had a baseline plasma HIV RNA level <1,000 copies/mL in the absence of ART. The median baseline plasma HIV RNA level using a standard assay (Abbott Real Time assay, lower limit of detection <40 copy/mL) was 77 copies/mL; 4/16 subjects had an “undetectable” (<40 copies/mL) baseline plasma HIV RNA level with this assay. The median baseline plasma HIV RNA level using an ultrasensitive “single copy assay” (lower limit of detection <0.3 copy/mL) was 23 copies/mL. The median baseline age was 49 years; most subjects (88%) were men. The median baseline CD4+ and CD8+ T cell counts were 616 and 897 cells/mm^3^, respectively; the median baseline CD4+ to CD8+ T cell count ratio was 0.71. The median nadir CD4+ T cell count was 590 cells/mm^3^. The median self-reported duration of known HIV diagnosis was 10 years.

Antiretroviral therapy was well tolerated and all subjects completed 24 weeks of ART. No significant adverse events occurred during the study. The majority of controllers (11/16) elected to continue ART after the 24-week study period. Five out of 16 subjects elected to discontinue ART after the 24-week study period at various times (median 10.0 weeks, interquartile range [IQR] 1.0 to 19.0 weeks) after the end of the treatment study. They have subsequently been followed for a median 63.0 (IQR 47.4 to 66.3) weeks after discontinuing ART, and at the time of last follow-up the plasma HIV RNA level using a standard assay (Abbott Real Time assay, lower limit of detection <40 copy/mL) was a median <40 (IQR<40 to 73) copies/mL. Of the 5 subjects who elected to discontinue ART after the end of the treatment study, 4/5 of the subjects had an “undetectable” pre-ART plasma HIV RNA level at baseline using a standard assay (Abbott Real Time assay, lower limit of detection <40 copy/mL).

### CD4+ T Cell Counts

**Figure 1:**
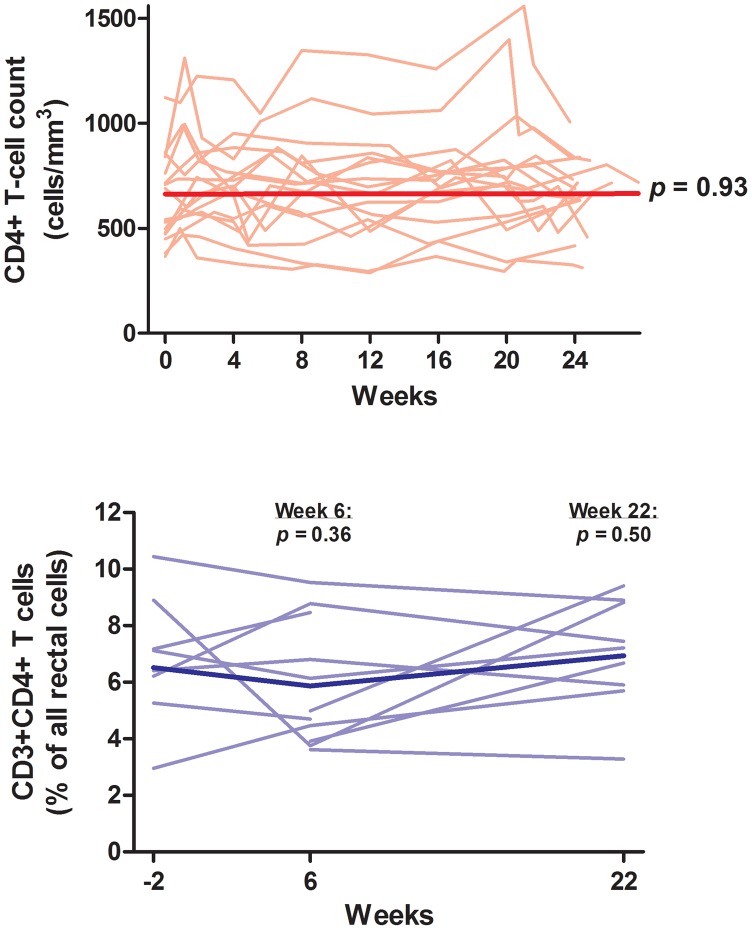
Change in peripheral CD4+ T cell count and rectal CD4+ T cell content. Thin lines indicate data for each individual subject. The thick line indicates the estimated mean value over time from mixed effects linear regression. P-values refer to change from baseline at each referenced time-point.

Controllers did not have a statistically significant increase in peripheral CD4+ T cell counts (mean 1.00-fold increase in CD4+ T cells at week 24, 95% confidence interval [CI] 1.05-fold decrease to 1.06-fold increase, p = 0.93) (Fig. 1A). Similarly, controllers did not have a statistically significant increase in %CD3+CD4+ T cells in the rectum (mean +0.4%, 95% CI −0.8% to 1.6%, p = 0.50) (Fig. 1B).

### Ultrasensitive Plasma HIV RNA and HIV Antibody Levels

**Figure 2:**
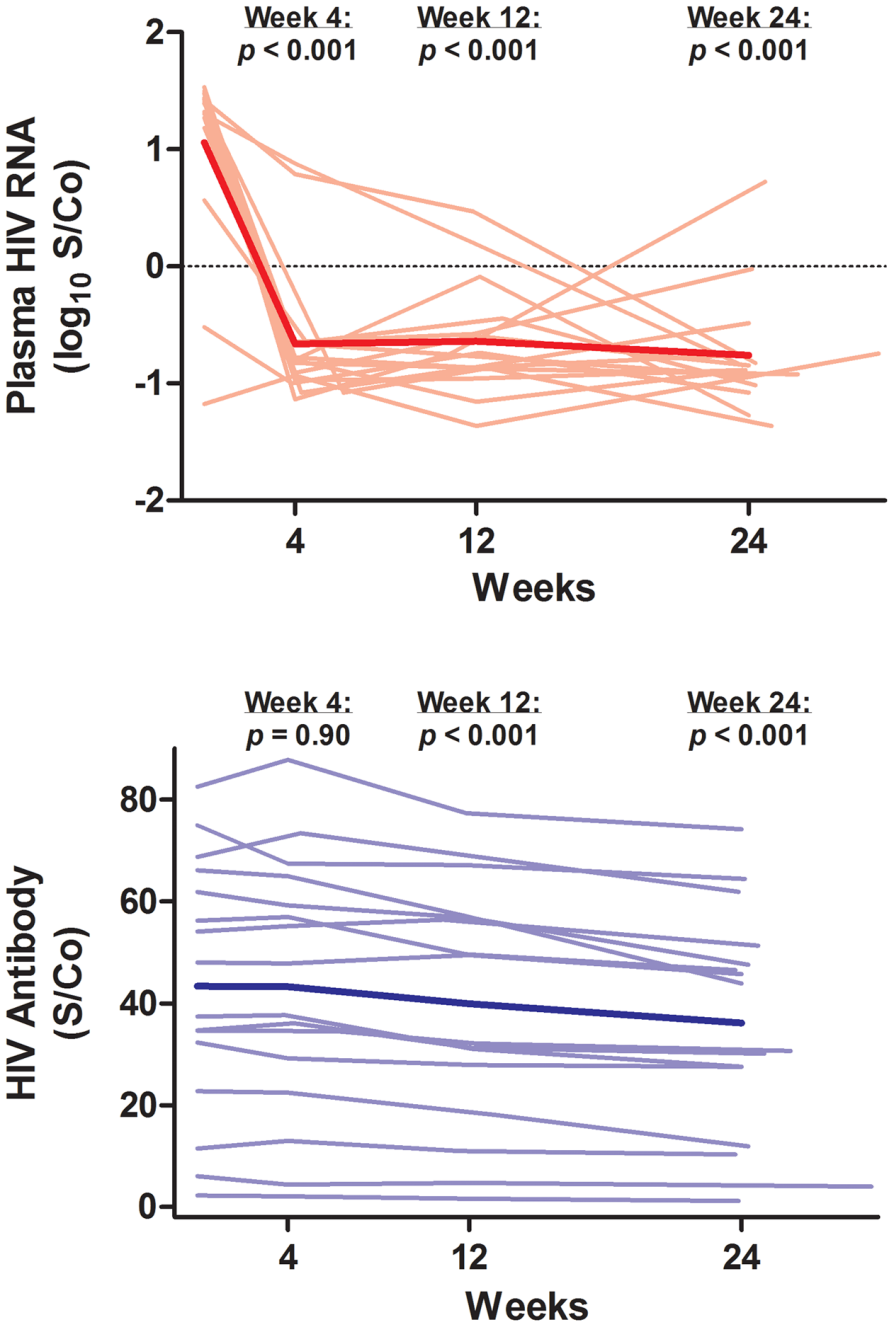
Change in ultrasensitive plasma HIV RNA and HIV antibody levels. Thin lines indicate data for each individual subject. The thick line indicates the estimated mean value over time from mixed effects linear regression. P-values refer to change from baseline at each referenced time-point. S/Co = signal/cutoff ratio.

Despite having low pre-ART plasma HIV RNA levels by conventional assays, controllers had an early and persistent decrease in ultrasensitive plasma HIV RNA levels after initiation of ART (mean 66-fold decrease in S/Co at week 24, 95% CI 155 to 28-fold decrease, p<0.001) (Fig. 2A). In addition, we examined change in HIV antibody levels as a surrogate measure of antigenic stimulation and viral persistence [10], [20], [21], [22], [23]. Controllers also had an early and persistent decrease in HIV antibody levels (mean −7.2 S/Co at week 24, 95% CI −9.6 to −4.8, p<0.001) (Fig. 2B).

### Cell-Associated HIV RNA and Total and Integrated HIV DNA

**Figure 3:**
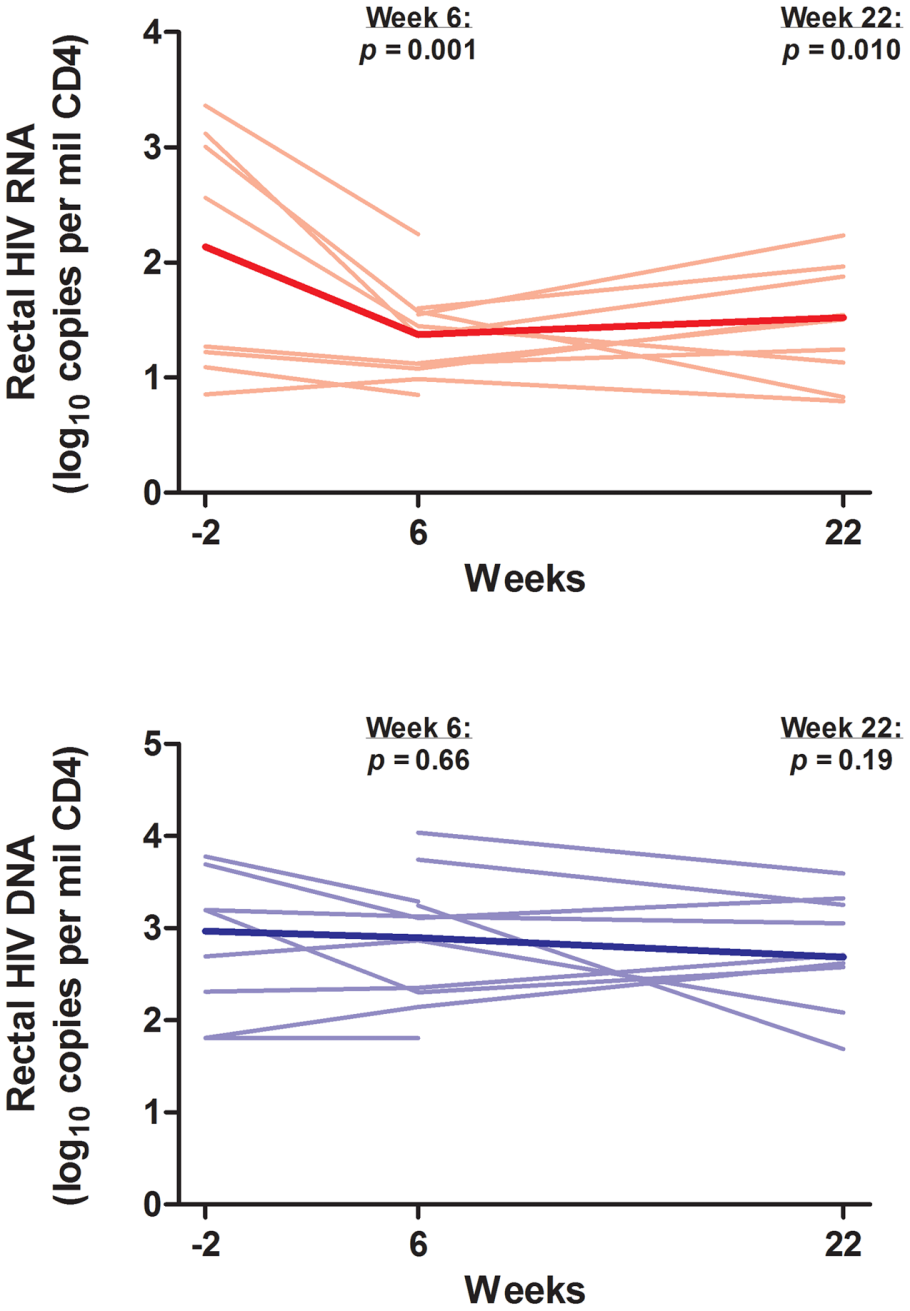
Change in cell-associated HIV RNA and total HIV DNA in rectum. Thin lines indicate data for each individual subject. The thick line indicates the estimated mean value over time from mixed effects linear regression. P-values refer to change from baseline at each referenced time-point.

**Figure 4:**
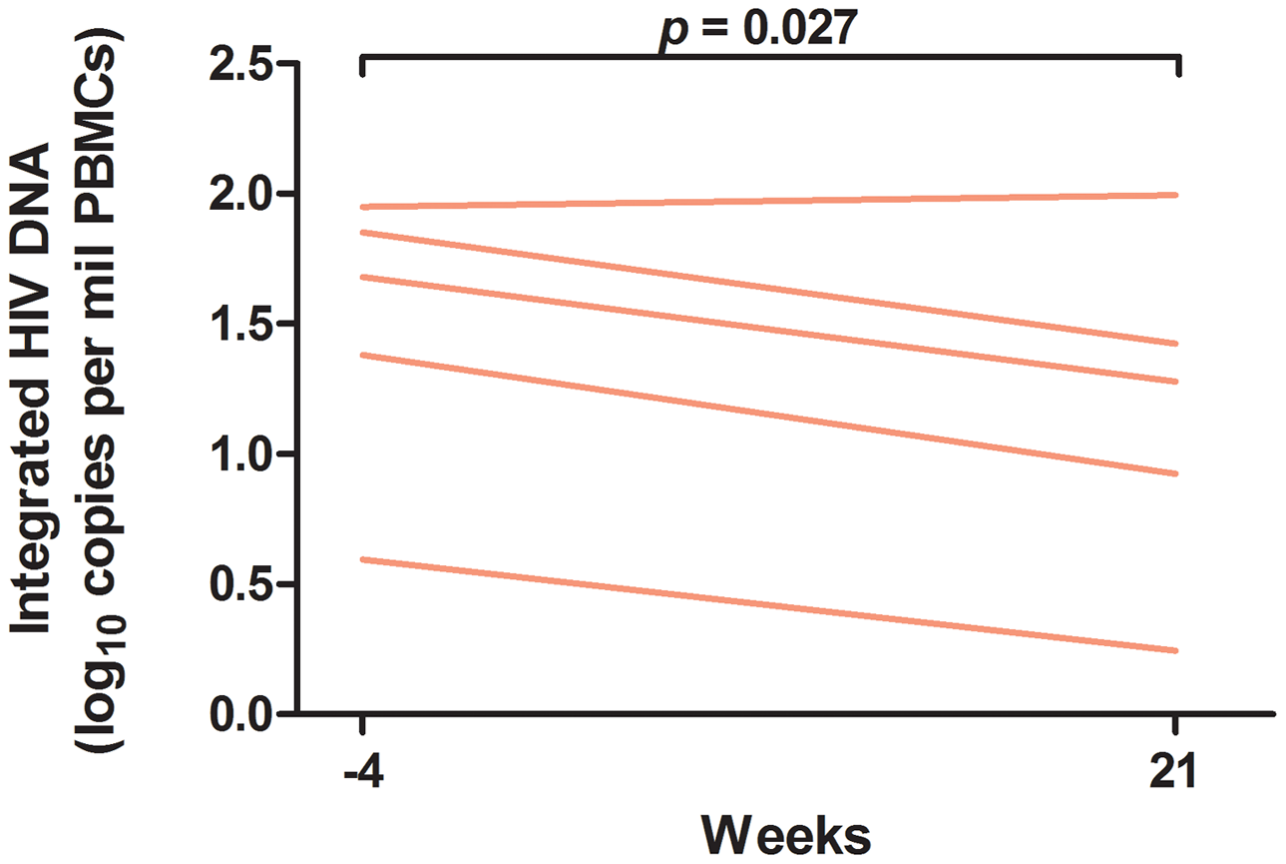
Change in integrated HIV DNA in peripheral blood. Change in integrated DNA levels before and after 21 weeks of antiretroviral therapy was measured using the paired t-test with bias-corrected and accelerated non-parametric confidence intervals. PBMCs = peripheral blood mononuclear cells.

At baseline, the median (IQR) levels of cell-associated HIV RNA and total HIV DNA in PBMCs were 6.9 (3.5, 45.7) S/Co per million CD4+ T cells and 57 (34, 138) copies/million CD4+ T cells, respectively. In PBMCs, controllers did not have a substantial decrease in cell-associated HIV RNA (mean 1.20-fold decrease in S/Co per million CD4+ T cells, 95% CI 2.4-fold decrease to 1.62-fold increase, p = 0.58) or total HIV DNA (mean 1.22-fold decrease in copies/million CD4+ T cells, 95% CI 1.95-fold decrease to 1.32-fold increase, p = 0.41) at week 24. However, controllers did have an early and persistent decrease in rectal cell-associated HIV RNA after initiation of ART, with a mean decrease of 0.61 log_10_ copies/million CD4+ cells, which corresponded to a 4.1-fold decrease (95% CI 12.0 to 1.40-fold decrease, p = 0.010) at week 22 (Fig. 3A). There was a similar trend towards a decrease in rectal total HIV DNA, with a mean decrease of 0.28 log_10_ copies/million CD4+ cells, which corresponded to a 1.91-fold decrease (95% CI 5.1-fold decrease to 1.38-fold increase, p = 0.19) at week 22 (Fig. 3B). We also measured integrated HIV DNA levels in PBMCs obtained through leukapheresis in 5 controllers. In these subjects, there was a statistically significant decrease in integrated HIV DNA after initiation of ART, with a mean decrease of 0.32 log_10_ copies/million PBMCs, which corresponded to a 2.1-fold decrease (95% CI 2.7 to 1.13-fold decrease, p = 0.027) at week 21 (Fig. 4).

### T Cell Activation/Dysfunction

**Figure 5:**
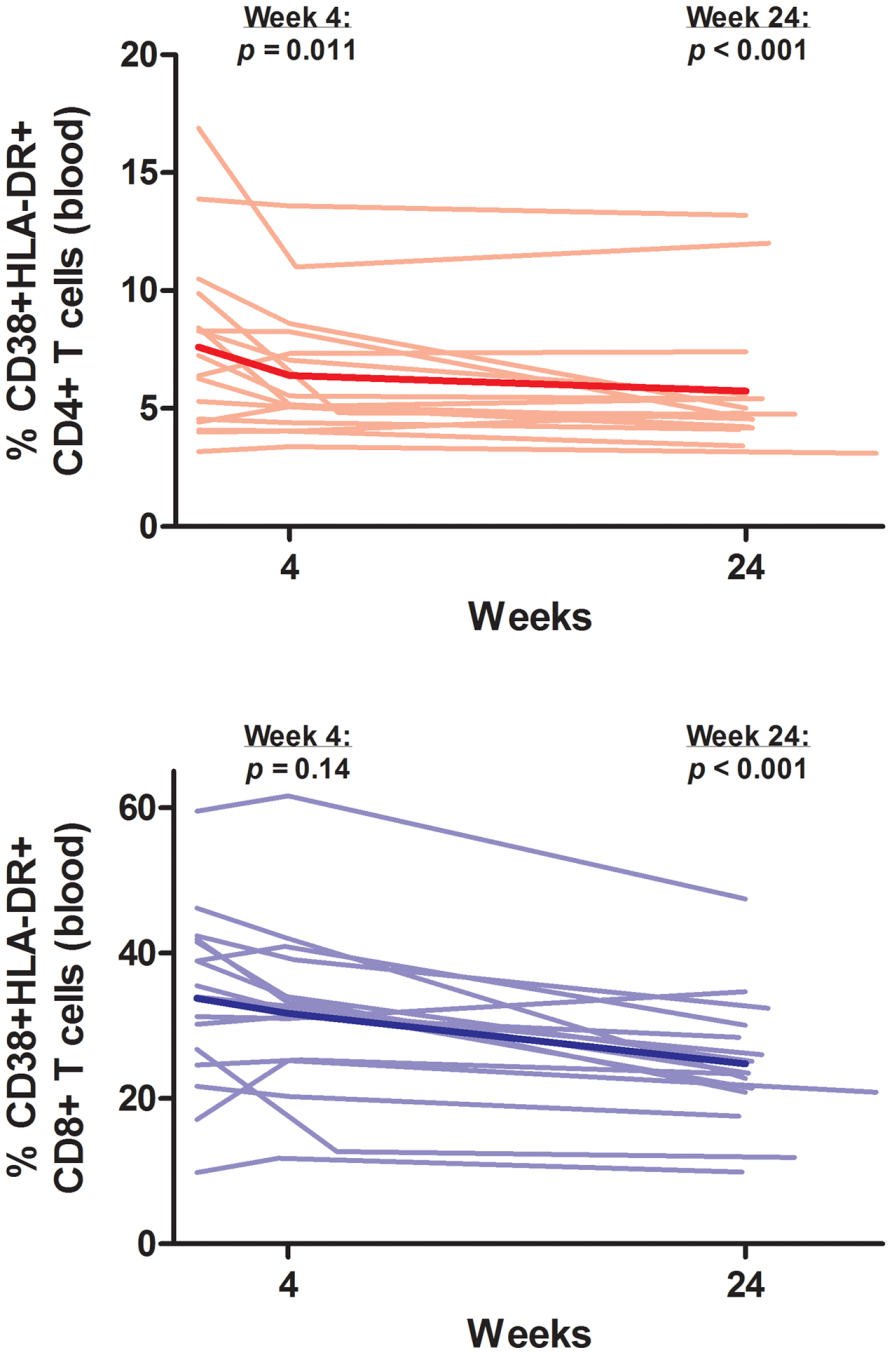
Change in T cell activation in peripheral blood. Thin lines indicate data for each individual subject. The thick line indicates the estimated mean value over time from mixed effects linear regression. P-values refer to change from baseline at each referenced time-point.

**Figure 6:**
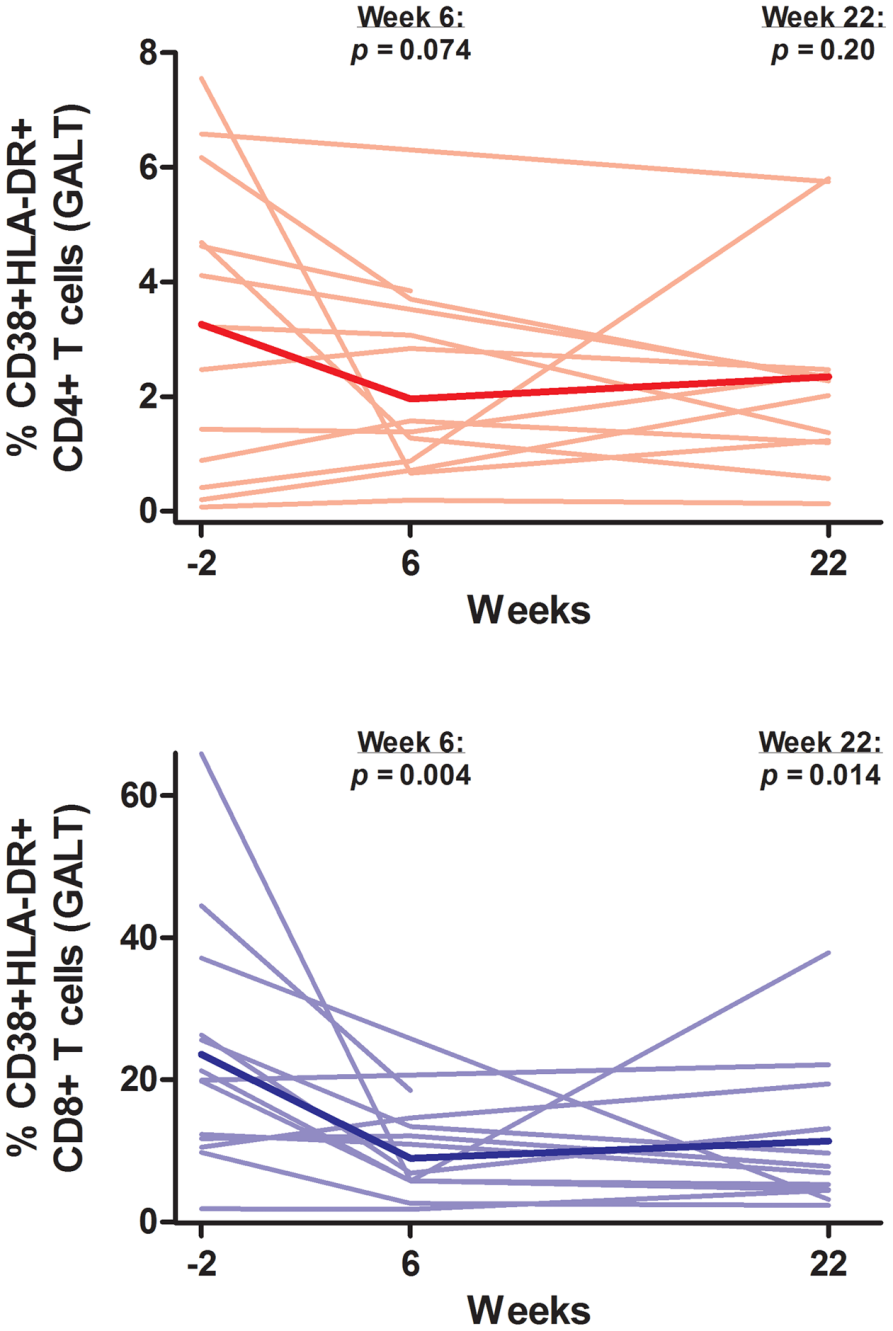
Change in T cell activation in rectum. Thin lines indicate data for each individual subject. The thick line indicates the estimated mean value over time from mixed effects linear regression. P-values refer to change from baseline at each referenced time-point. GALT = gut-associated lymphoid tissue.

Markers of T cell activation/dysfunction in blood and gut also decreased substantially with ART. In PBMCs, controllers had a mean decrease of 1.9% in %CD38+HLA-DR+ CD4+ T cells (95% CI −2.8% to −0.9%, p<0.001) and a mean decrease of 9.0% in %CD38+HLA-DR+ CD8+ T cells (95% CI −12.4% to −5.6%, p<0.001) at week 24 (Fig. 5). Controllers also had a mean decrease of 1.6% in %PD-1+ CD4+ T cells (95% CI −3.1% to −0.1%, p = 0.04) and a mean decrease of 4.5% in %PD-1+ CD8+ T cells (95% CI −6.4% to −2.6%, p<0.001) in PBMCs at week 24. In the rectum, controllers had a trend towards a decrease in %CD38+HLA-DR+ CD4+ T cells (mean −0.9%, 95% CI −2.3% to +0.5%, p = 0.20) and a statistically significant mean decrease of 12.2% in %CD38+HLA-DR+ CD8+ T cells (95% CI −21.9% to −2.5%, p = 0.014) at week 22 (Fig. 6).

### Plasma Biomarkers

**Figure 7:**
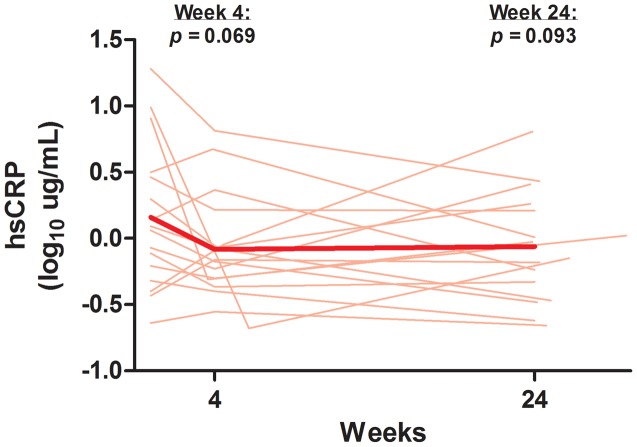
Change in high sensitivity C-reactive protein. Thin lines indicate data for each individual subject. The thick line indicates the estimated mean value over time from mixed effects linear regression. P-values refer to change from baseline at each referenced time-point. hsCRP = high sensitivity C-reactive protein.

At baseline, the median (IQR) levels of high sensitivity C-reactive protein (hsCRP), interleukin-6 (IL-6), soluble CD14 (sCD14), and D-dimer were 1.20 (0.55, 3.03) ug/mL, 1.71 (1.28, 4.42) pg/mL, 1696 (1446, 1971) ng/mL, and 0.37 (0.28, 0.56) ug/mL, respectively. After ART initiation, there was a trend towards a decrease in hsCRP, with a mean 1.74-fold decrease (95% CI 3.2-fold decrease to 1.04-fold increase, p = 0.069) at week 4, and a mean 1.67-fold decrease (95% CI 3.0-fold decrease to 1.09-fold increase, p = 0.093) at week 24 (Fig. 7). We also observed similar trends in IL-6 (mean 1.34-fold decrease, 95% CI 2.1-fold decrease to 1.19-fold increase, p = 0.22), sCD14 (mean −44.2, 95% CI −138.6 to +50.3, p = 0.36), and D-dimer (mean 1.30-fold decrease, 95% CI 2.1-fold decrease to 1.25-fold increase, p = 0.29) levels after 24 weeks of ART, although these trends did not reach statistical significance.

### HIV-Specific T Cell Responses

At baseline, the median (IQR) levels of percentage of Gag-specific IFNγ+IL2+ CD4+ and CD8+ T cell responses in PBMCs were 0.10% (0.03%, 0.17%) and 1.53% (0.37%, 2.55%), respectively. Controllers did not have a substantial change in the percentage of Gag-specific IFNγ+IL2+ CD4+ T cell responses in PBMCs (mean 1.07-fold decrease, 95% CI 1.40-fold decrease to 1.22-fold increase, p = 0.62), although there was a trend towards a decrease in the percentage of Gag-specific IFNγ+IL2+ CD8+ T cell responses in PBMCs (mean 1.28-fold decrease, 95% CI 1.68-fold decrease to 1.03-fold increase, p = 0.075) at week 24. At baseline, the median (IQR) levels of percentage of total (IFNγ, IL-2, TNFα, and/or CD107a) Gag-specific CD4+ or CD8+ in the rectum were 0.56% (0%, 1.3%) and 0.22% (0.08%, 0.32%), respectively. Controllers did not have a substantial change in the percentage of total Gag-specific CD4+ (mean 1.26-fold increase, 95% CI 1.82-fold decrease to 2.9-fold increase, p = 0.58) or CD8+ (mean 1.61-fold decrease, 95% CI 3.6-fold decrease to 1.37-fold increase, p = 0.24) T cell responses in the rectum at week 22.

### “Elite” Controllers

**Figure 8:**
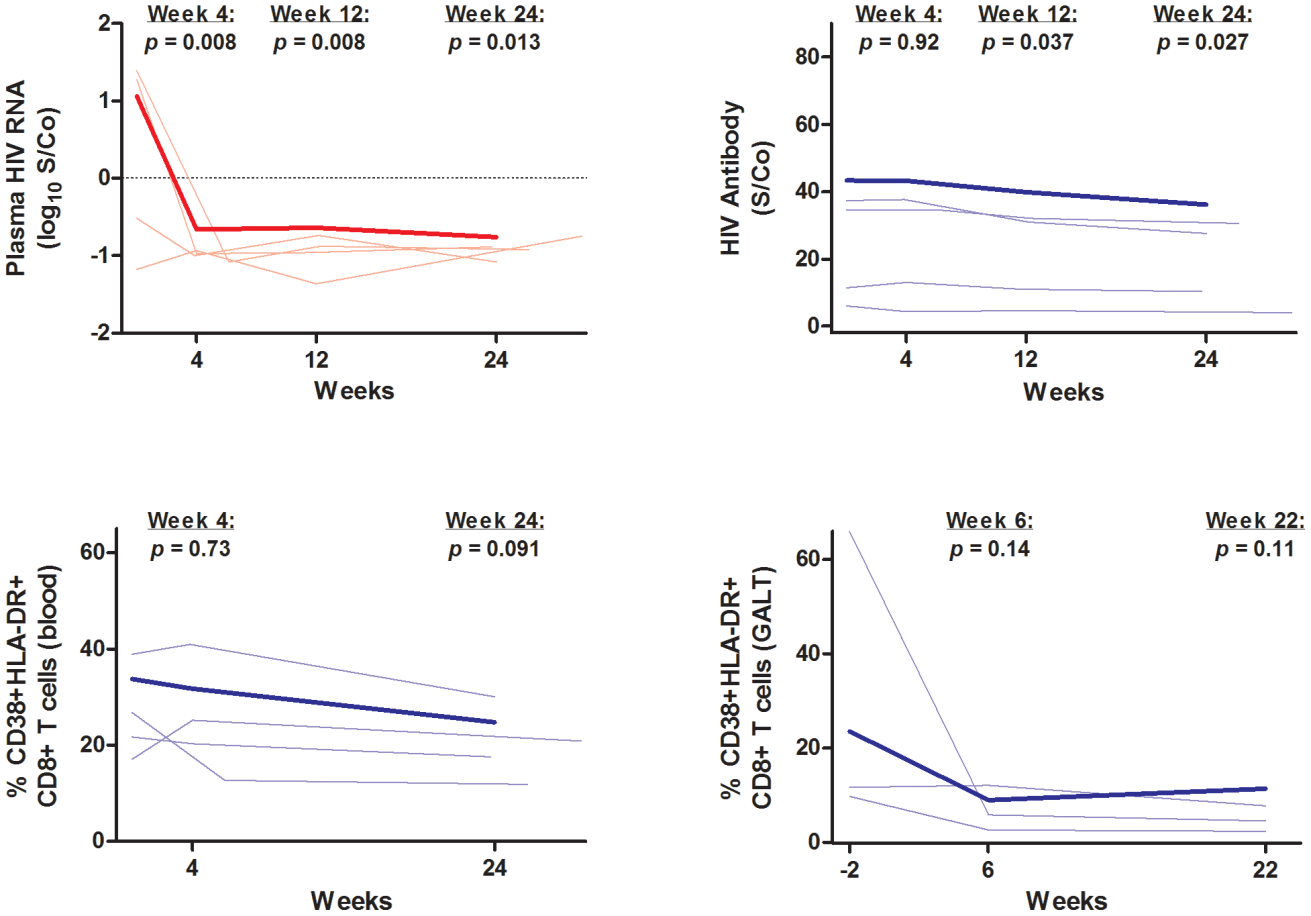
“Elite” controllers (n = 4). Change in ultrasensitive plasma HIV RNA, HIV antibody levels, and T cell activation in peripheral blood and rectum in 4/16 controllers with baseline plasma HIV RNA <40 copies/mL. Thin lines indicate data for each individual subject (n = 4). The thick line indicates the estimated mean value over time from mixed effects linear regression for the entire cohort (n = 16). The y-axes are on the same scale as Figures 2, 5, and 6. P-values refer to change from baseline at each referenced time-point. S/Co = signal/cutoff ratio. GALT = gut-associated lymphoid tissue.

At baseline, 4/16 controllers had an “undetectable” pre-ART plasma HIV RNA level using a standard assay (Abbott Real Time assay, lower limit of detection <40 copy/mL). Despite having this extremely low pre-ART plasma HIV RNA level, this subset of so-called “elite” controllers had a statistically significant decrease in ultrasensitive plasma HIV RNA levels after initiation of ART (mean 14-fold decrease in S/Co at week 24, 95% CI 115 to 1.74-fold decrease, p = 0.013) (Fig. 8A). Similarly, this subset of controllers had a statistically significant decrease in HIV antibody levels (mean −4.2 S/Co at week 24, 95% CI −7.9 to −0.5, p = 0.027) (Fig. 8B). Finally, we observed similar trends in immune activation in these 4 controllers after initiation of ART. There was a mean decrease of 6.0% in %CD38+HLA-DR+ CD8+ T cells in PBMCs at week 24 (95% CI −13.0% to +0.10%, p = 0.091, Fig. 8C) and a mean decrease of 24.3% in %CD38+HLA-DR+ CD8+ T cells in the rectum at week 22 (95% CI −54.1% to +5.6%, p = 0.11, Fig. 8D).

## Discussion

In this first prospective study of antiretroviral therapy initiation in asymptomatic HIV-infected controllers, 24 weeks of ART was safe and well-tolerated. Despite being able to maintain very low plasma HIV RNA levels in the absence of ART, controllers had readily measurable levels of HIV RNA and DNA in the gut. Antiretroviral therapy led to statistically significant decreases in ultrasensitive plasma HIV RNA levels, HIV antibody levels, rectal cell-associated HIV RNA, and immune activation in the blood and gut. Collectively, these data suggest that HIV in most controllers is replication-competent [24], [25], [26], and that host rather than virologic factors account for the remarkable degree of viral control in these unique individuals. We also observed a statistically significant decrease in levels of integrated HIV DNA with ART, while total HIV DNA levels remained stable. These findings may be due to an excess of unintegrated HIV DNA in controllers, as previously reported by our group [27].

We observed that measures of immune activation/dysfunction decreased as measures of virologic burden and HIV antigenic stimulation decreased with ART. We also observed a trend towards a decrease in hsCRP (a measure of systemic inflammation) with ART; similar trends were observed with IL-6, sCD14, and D-dimer. These biomarkers have been shown to be strong, consistent, and independent predictors of increased morbidity and mortality in HIV infection [18], [19], [28]. Because the confidence intervals were wide, however, we cannot assess with certainty whether the observed decrease in hsCRP levels has any clinical relevance; it would be important to pursue these findings in future, larger studies. Taken together, however, these data suggest that there may be immunologic consequences to even very low levels of viral replication. This latter finding may have important implications for HIV-infected non-controllers as well [29], [30], [31], [32], [33].

Importantly, our study also shifts the field's working definition of a “functional cure.” On one hand, our data suggest that a complete block of viral replication is not necessary to achieve long-term virologic control. However, natural long-term virologic control appears to be coming at an immunologic and/or clinical “cost,” at least as defined by increased levels and manifestations of immune activation. Thus, although further study of controllers is warranted, untreated HIV-infected controllers may not represent the best model of a functional cure, if we believe that a cure should require a disease-free (and not just treatment-free) state.

Several limitations of our study deserve comment. First, this was a small pilot study and our findings should be replicated in a larger study of HIV-infected controllers with a longer duration of follow-up. In our study of controllers who had relatively high baseline CD4+ T cell counts, 24 weeks of ART did not appear to confer a CD4+ T cell count benefit. In studies of HIV-infected non-controllers, a greater absolute decrease in plasma HIV RNA levels during the early period after ART initiation has been shown to be a consistent predictor of an early increase in CD4+ T cell counts (with much of the increase assumed to be due to redistribution) [34]. In our study, although we did observe a significant decrease in ultrasensitive plasma HIV RNA levels with initiation of ART, the absolute change was small compared to that observed in HIV-infected non-controllers; this may have partially accounted for the limited changes in peripheral CD4+ T cell counts. It is possible that with a much longer duration of follow-up, an increase in CD4+ T cell count may have been observed. Second, there was a trend towards a decrease in the percentage of Gag-specific IFNγ+IL2+ CD8+ T cell responses in PBMCs, although a similar trend was not observed in the rectum. This observation raises the possibility that initiation of ART in controllers may reduce host mechanisms of virologic control, leading to rebound in viremia if ART is discontinued. However, in the 5 subjects who elected to discontinue ART after the 24-week study period, there was no evidence of rebound in plasma viremia after discontinuation of ART. Nevertheless, the long-term safety of ART in controllers should be confirmed. Third, we enrolled a relatively heterogeneous group of controllers. As we and others have shown, however, controllers are a heterogeneous group with varying levels of steady-state viremia; there appears to be a continuum of viremia across controllers [1], [10], [12], . In order to determine whether there is a differential effect of ART on a spectrum of controllers, we enrolled individuals whose baseline plasma HIV RNA levels spanned from nearly 0 to 1000 copies RNA/mL for at least 12 months (median duration of HIV diagnosis 10 years, IQR 4.5 to 24 years). Thus, our study included controllers who had both low-level but detectable and undetectable pre-ART plasma HIV RNA levels using conventional assays. Remarkably, however, even amongst the latter group of “elite” controllers who had undetectable pre-ART plasma HIV RNA levels at baseline, we observed a statistically significant decrease in ultrasensitive plasma HIV RNA levels and HIV antibody levels, and a trend towards a decrease in immune activation with ART. Fourth, although 24 weeks of ART significantly decreased levels of CD4+ and CD8+ T cell activation, it did not normalize them to levels observed in HIV-uninfected individuals [41]. Thus, at least in HIV-infected controllers, low-level viral replication is unlikely to be the only factor contributing to immunologic disease. The potential role of other factors that might contribute to immune activation—including co-infections and substance abuse—could not be addressed in this pilot study, but might be addressed in future studies with larger cohorts. It would also be important to systematically assess the individual and potentially synergistic contributions of ART and lifestyle modifications towards decreasing inflammation, immune activation, and clinical disease in HIV-infected controllers [14]. Finally, it is worth noting that there may be multiple pathways to virologic control, some of which may represent an appropriate model of a “functional cure” and may not receive an additional benefit from ART.

In summary, 24 weeks of ART was safe and well-tolerated in chronically HIV-infected controllers. Antiretroviral therapy in controllers led to significant decreases in ultrasensitive plasma and rectal HIV RNA, HIV antibody levels, and markers of immune activation/dysfunction in blood and gut, confirming that HIV replication persists in controllers and contributes to a chronic inflammatory state. We acknowledge that this was a small pilot study and that our findings would be ideally replicated in a larger, randomized, clinical-endpoint study. However, the relative rarity of HIV-infected controllers may make such a study impractical, if not impossible. In the absence of such a study, clinicians will need to weigh the potential benefits of ART (suggested by the changes in immune activation and biomarkers observed in our study) with the potential risks and costs associated with long-term antiretroviral therapy.

## Materials and Methods

### Ethics Statement

All subjects provided written informed consent. This study was approved by the University of California San Francisco (UCSF) Committee on Human Research.

### Ultrasensitive Plasma HIV RNA

The isothermal Transcription Mediated Amplification (TMA) assay (Aptima, Gen-Probe/Hologic) was used to measure ultrasensitive plasma HIV RNA levels at weeks 0, 4, 12, and 24. This is a nucleic acid-amplification test that has been FDA-approved for the early detection of HIV infection in blood donors [42], [43], [44]. It is a highly specific and sensitive assay, with a singlicate 50% detection limit of 3.6–14 copies/mL [45], [46]. The assay was performed in triplicate on 0.5 mL plasma (1.5 mL total plasma), improving the overall 50% detection limit to <5 copies/mL. The output is a signal/cutoff (S/Co) ratio (range 0–30), with S/Co<1.0 = “negative” and S/Co≥1.0 = “positive.” Ultrasensitive plasma HIV RNA levels were also measured at weeks 0 and 12 with a “single copy assay” (lower limit of detection <0.3 copy/mL), using a median 7.3 mL of plasma [47].

### Plasma HIV Antibody Levels

A “de-tuned” or less-sensitive enzyme immunoassay (LS-VITROS) was used to measure HIV antibody levels at weeks 0, 4, 12, and 24. The VITROS (Ortho-Clinical Diagnostics) is an FDA-approved diagnostic assay for the detection of IgM/IgG antibodies to HIV-1/-2. The less-sensitive modification tests 1∶400 dilutions of plasma and calculates a S/Co ratio (range 0–80), and has been validated as a method to identify early HIV infection [48].

### Cell-Associated HIV RNA and Total HIV DNA (PBMCs)

Cell-associated HIV RNA and total HIV DNA were measured from PBMCs at weeks 0, 4, and 24. Cell-associated HIV RNA was measured using modifications of published methods (Aptima, Gen-Probe/Hologic) [10], [49]. The output is a S/Co ratio (range 0–30), with S/Co<1.0 = “negative” and S/Co≥1.0 = “positive.” All S/Co ratios were normalized to per million CD4+ T cells. Total HIV DNA was measured using modifications of published methods with an overall sensitivity of 1 copy/3 µg of DNA (450,000 PBMCs) [10], [50], [51], [52]. All total HIV DNA levels were normalized to per million CD4+ T cells.

### Integrated HIV DNA (PBMCs)

Integrated HIV DNA was measured from PBMCs at weeks −4 and 21. DNA was prepared (Qiagen Mid) and integrated HIV DNA was measured using a published repetitive sampling method because integration levels are known to be low in controllers [27], [53]. At least 42 Alu-gag PCR reactions were performed with 150,000 diploid genomes per PCR, for a total of 6.3 million diploid genomes assayed per subject.

### T Cell Immunophenotyping and Cytokine Flow Cytometry (PBMCs)

PBMCs were isolated from whole blood, cryopreserved, and stored at the UCSF AIDS Specimen Bank. Markers of T cell activation/dysfunction and antigen-specific T cell responses were measured at weeks 0, 4, and 24 at the UCSF Core Immunology Laboratory, using published methods that have been optimized and validated for cryopreserved PBMCs [54]. Briefly, cryopreserved PBMCs were rapidly thawed in warm media, counted on an Accuri C6 (BD Biosciences) with the Viacount assay (Millipore), and washed and stained the same day (T cell immunophenotyping) or rested overnight (cytokine flow cytometry [CFC]). The average viability of thawed cells was 93% (range 61–98%; 80% of samples had viability >90%).

For T cell immunophenotyping, the percent of activated (CD38+/HLA-DR+/PD1+) CD4+ and CD8+ T cells were measured; these markers of immune activation/dysfunction have been shown to be strong and independent predictors of HIV disease progression [12], [41], [55], [56], [57]. Cells were stained with Aqua Amine Reactive Dye (AARD, Invitrogen) to discriminate dead cells, washed, and stained with fluorescently-conjugated monoclonal antibodies: CD3-Pacific Blue (BD Pharmingen), CD38-PE, HLA-DR-FITC, PD1- Alexa647 (BD Biosciences), CD4-PE Texas Red, and CD8-QDot 605 (Invitrogen). In each experiment a fluorescent-minus one control was included for CD38, HLA-DR, and PD-1. Stained cells were washed, fixed in 0.5% formaldehyde (Polyscience), and held at 4C until analysis.

For CFC, rested PBMCs were stimulated for 18–22 h at 37C with overlapping peptide pools corresponding to HIV-1 Con B Gag peptides (NIH 8117) in the presence of 0.5 ug/mL Brefeldin A and 0.5 ug/mL Monensin (Sigma-Aldrich). A control well with no stimulation was run in parallel for each sample. Cells were washed and stained with AARD, fixed, and permeabilized for intracellular staining with antibodies against CD3-Pacfic Blue, IFNγ-FITC, IL-2-PE (BD BioScience), CD4-PE Texas Red, and CD8-QDot 605 (Invitrogen). Cells were washed and stored at 4C until analysis. We focused on Gag-specific IFNγ+IL2+ T cell responses given that we have shown that these responses are associated with control of HIV replication in controllers [5], [35], [58].

Stained cells were run on a customized BD LSR II (BD Bioscience). 100,000 and 500,000 lymphocytes were collected for immunophenotyping and CFC samples, respectively. Data were compensated and analyzed using FlowJo (Tree Star) to determine the proportion of CD4+ and CD8+ T cells expressing each of the T cell or cytokine markers. Combinations of markers were calculated in FlowJo using the Boolean gate function. For CFC data, results from control wells with no stimulation were subtracted from stimulated results.

### Plasma Biomarkers

High sensitivity C-reactive protein (hsCRP), interleukin-6 (IL-6), soluble CD14 (sCD14), and D-dimer levels were measured on stored fasting plasma samples at weeks 0, 4, and 24 at the Laboratory for Clinical Biochemistry Research at the University of Vermont. hsCRP was measured with a BN II nephelometer (Siemens Diagnostics, Deerfield, IL), IL-6 was measured with Chemiluminescent Sandwich enzyme-linked immunosorbent assay, sCD14 with a standard ELISA (both R&D Systems, Minneapolis, MN), and D-dimer was measured with an immunoturbidometric method on the Sta-R analyzer, Liatest D-DI (Diagnostica Stago, Parsippany, NJ). Interassay coefficients of variation for a number of different control materials of different values averaged ∼10% or less for all assays.

### Gut-Associated Lymphoid Tissue (GALT)

Thirty colorectal biopsy specimens were obtained 10–20 cm from the anal verge using 3 mm jumbo forceps at weeks −2, 6, and 22. Eighteen to 24 biopsy pieces were placed into 10 mL RPMI-1640 media containing fetal calf serum (15%), penicillin (100 U/mL), streptomycin (100 ug/mL), and L-glutamine (2 mM). Fresh colorectal cells were isolated on the same day using a modification of a published protocol designed to optimize yield and viability of mucosal lymphocytes without compromising the detection of most surface antigens [59]. Briefly, biopsy pieces underwent two rounds of digestion in 0.5 mg/mL collagenase type II (Sigma-Aldrich). Each digestion was followed by disruption of the tissue with a syringe bearing a 16-gauge blunt end needle and subsequent passage through a 70 µm cell strainer. Yields were 9.5–31×10^6^ (mean 18×10^6^) total rectal cells. One aliquot of cells was set aside for flow cytometry and stained with CD45-FITC, CD3-APC and CD4-PE (BD biosciences) for 15 min at 25C. Propidium iodide was added to stain non-viable cells and samples were run on an Accuri C6 to determine the total number of viable mononuclear cells and proportion and absolute number of viable CD45+ leukocytes and CD4+ T cells. Another aliquot of cells was frozen at −80C for subsequent nucleic acid extraction.

### Cell-Associated HIV RNA and Total HIV DNA (GALT)

Total HIV RNA was measured from rectal cells using a published method [40]. Three replicates of up to 500 ng RNA were assayed for total processive HIV RNA transcripts using primers (HXB2 positions 522–543, 626–643) and probe (559–584) from the LTR region [60]. Genomic HIV RNA standards (2.5×10^0^ to 2.5×10^5^) were prepared from lab stocks of NL4-3 virions by extracting and quantifying HIV RNA using the Abbot Real Time assay. HIV RNA copy numbers were normalized to cellular input into the PCR, as determined by RNA mass (assuming 1 ng RNA = 1000 cells [61]), which has been shown to correlate with levels of GAPDH RNA [62]. Results were further normalized by the percent of cells that were CD3+CD4+ by flow cytometry and expressed as copies/10^6^ CD4+T cells.

Total HIV DNA was measured from rectal cells using a published method [40]. Three replicates of up to 500 ng DNA were assayed for HIV DNA using a modification of a published TaqMan PCR assay that uses primers/probe from the LTR region (as above). External standards (10^5^ to 1) were prepared from DNA extracted from known numbers of 8E5 cells (NIH AIDS Reagent Program), each of which contains one integrated HIV genome per cell. HIV DNA copy numbers were normalized to cellular input into the PCR, as determined by DNA mass (assuming 1 ug DNA = 160,000 cells). Results were further normalized by the percent of cells that were CD3+CD4+ by flow cytometry and expressed as copies/10^6^ CD4+T cells.

### T Cell Immunophenotyping and Cytokine Flow Cytometry (GALT)

Markers of T cell activation (CD38+/HLA-DR+) and total Gag-specific responses (Gag-specific CD4+ and CD8+ T cells expressing one or more of IFNγ, IL-2, TNFα, and/or CD107a [63], [64], [65]) were measured from rectal cells at weeks −2, 6, and 22. We focused on these responses given that we have shown that these mucosal T cell responses are associated with control of HIV replication in controllers [36].

For T cell immunophenotyping of freshly isolated rectal cells, similar methods were used as for PBMCs [59]. For CFC, freshly isolated rectal cells were rested overnight at 37C, 5%CO2, in R15 containing 0.5 mg/mL piperacillin-tazobactam, then similar methods were used as for PBMCs [59]. To account for the lower numbers of events and elevated baseline cytokine staining in mucosal samples, response data from peptide-stimulated wells were first compared against unstimulated controls using a published algorithm to determine statistical significance, prior to background subtraction [36], [66].

### Statistical Methods

Mixed effect linear models with random slopes and intercepts were used to examine change in virologic and immunologic measurements over time. Changes in integrated HIV DNA levels were assessed by estimating the mean change and its bias-corrected and accelerated non-parametric confidence intervals, and using a paired t-test to obtain a corresponding p-value [67]. All statistical analyses were conducted with Stata version 11.1 (Stata Corp).
